# Water aerobics II: maternal body composition and perinatal outcomes after a program for low risk pregnant women

**DOI:** 10.1186/1742-4755-6-1

**Published:** 2009-01-06

**Authors:** Sergio R Cavalcante, Jose G Cecatti, Rosa I Pereira, Erica P Baciuk, Ana L Bernardo, Carla Silveira

**Affiliations:** 1 Department of Obstetrics and Gynecology, School of Medical Sciences, University of Campinas (UNICAMP), Sao Paulo, Brazil; 2 Department of Anesthesiology, School of Medical Sciences, University of Campinas (UNICAMP), Sao Paulo, Brazil

## Abstract

**Background:**

To evaluate the effectiveness and safety of water aerobics during pregnancy.

**Methods:**

A randomized controlled trial carried out in 71 low-risk sedentary pregnant women, randomly allocated to water aerobics or no physical exercise. Maternal body composition and perinatal outcomes were evaluated. For statistical analysis Chi-square, Fisher's or Student's t-tests were applied. Risk ratios and their 95% CI were estimated for main outcomes. Body composition was evaluated across time using MANOVA or Friedman multiple analysis.

**Results:**

There were no significant differences between the groups regarding maternal weight gain, BMI or percentage of body fat during pregnancy. Incidence of preterm births (RR = 0.84; 95%CI:0.28–2.53), vaginal births (RR = 1.24; 95%CI:0.73–2.09), low birthweight (RR = 1.30; 95%CI:0.61–2.79) and adequate weight for gestational age (RR = 1.50; 95%CI:0.65–3.48) were also not significantly different between groups. There were no significant differences in systolic and diastolic blood pressure and heart rate between before and immediately after the water aerobics session.

**Conclusion:**

Water aerobics for sedentary pregnant women proved to be safe and was not associated with any alteration in maternal body composition, type of delivery, preterm birth rate, neonatal well-being or weight.

## Background

The association between good health and physical activity is not recent. Studies report that both the public in general and scientists and physicians in particular are concerned with the regular practice of moderate physical exercise, which is believed to contribute towards the length and quality of life [1-3]. Today, different age groups, genders, ethnic groups and especially pregnant women are concerned with physical well-being, and as there were no data in humans to justify that exercise should be limited during pregnancy, this explains, in part, why exercise programs for pregnant women are becoming increasingly popular [4].

During pregnancy, considerable anatomical and physiological changes take place in the expectant mother, including weight gain, and these changes are necessary for the development of the fetus [5-7]. However, the increase in weight may sometimes be excessive, in which case it becomes a risk factor for the pregnancy, requiring nutritional support and possibly a guided program of physical exercise and prenatal care [3-7]. Changes also occur in the pregnant woman's posture and gait as a result of the increase in lumbar lordosis, resulting in lower back pain that appears to improve following physical activity in water [3,8-10].

Today, the practice of physical activity is recommended as part of a healthy pregnancy [3,4,11]. This subject is controversial among investigators and obstetricians, since the association between physical activity and pregnancy is not always positive, involving the possibility of prematurity and low birth weight [12-14]. There is still much to be learned with respect to the intensity, timing and duration of regular physical activity and whether the consequent physiological changes in the expectant mother and her child are indeed beneficial. Further studies are required to clarify these issues [6,15].

The concerns regarding the benefits of physical exercise during pregnancy are related to the child's birthweight, maternal and fetal cardiac changes, risk of abortion and preterm delivery, and of injury due to increased maternal weight [11]. Another concern is whether the pregnant woman should carry out land- or water-based exercise. Katz [2] reported that water-based physical exercise increases the extracellular fluid in the vascular spaces, producing an increase in central blood volume caused by the hydrostatic force of the water. In addition, water-based exercise results in lower impact and less risk of traumatic lesions to which a pregnant woman is particularly vulnerable. Several authors have also reported that the practice of water-based physical exercise may be used as a form of treatment for reducing edema [9,13,16]. On the other hand, Kramer [14] reported that ground-based aerobics during pregnancy may improve the physical fitness level of the expectant mother. However, there is no evidence yet to completely eliminate the possibility of risks to the mother or the fetus [11].

Another concern is related to the intensity of the practice of physical exercise, particularly land-based exercise, since the expectant mother may be subject to any of the aforementioned risks [17]. Although there are studies that report the benefits of the practice of moderate, ground-based physical activity for pregnant women, there are few research studies on the practice of moderate, water-based physical activity for pregnant women, the benefits to the mother and the fetus, and the perinatal benefits [18,19].

There has currently been an increase in the number of pregnant women interested in carrying out physical exercise during pregnancy, particularly water-based exercise. More studies are therefore required to clarify the benefits and the risks of these activities, both for the expectant mother and for the fetus. The objective of this study was to evaluate the effectiveness and safety of a program of water aerobics for low risk, sedentary pregnant women on the evolution of pregnancy, maternal body composition and perinatal outcomes.

## Methods

An open, randomized controlled clinical trial was carried out and its methods are described in detail elsewhere [20]. The minimum sample size for the present study was calculated at 34 pregnant women in each group. This was based on a study by Prevedel et al. [19], in which the evolution of the body composition of pregnant women carrying out water aerobics was compared with that of a group of sedentary expectant mothers, showing a percentage of body fat at the end of pregnancy of 29.2 ± 4.4, and establishing a desired minimum difference between the groups of 3%, with a type I error of 0.05 and a type II error of 0.20. Seventy-one expectant mothers were enrolled between March 2002 and November 2004.

Low-risk, sedentary pregnant women between 16–20 weeks of gestation were selected from among those attending the prenatal outpatient clinic of the institution or at a neighboring basic healthcare center. Women with a history of two or more Cesarean sections, those with medical conditions contraindicating the practice of physical exercise and/or with some practical impediment to participating in the program were excluded from the study. Women fulfilling admission criteria were identified at the beginning of their prenatal care and invited to participate in a meeting with one of the investigators. At this meeting, the expectant mothers received information on the objectives of the study, as well as an explanation of the evaluations and protocols that had to be carried out. Those who agreed to participate in the study signed an informed consent form and filled out an admission questionnaire. The study was approved by the local Institutional Review Board prior to initiation.

The volunteers were randomly allocated to one of two groups according to admission order and following a computer generated randomization list. To guarantee the concealment for the randomization procedure, each sequential number corresponded to a sealed opaque envelope containing the questionnaires and information regarding the randomization group: Water Aerobics Group, women who would practice water aerobics regularly throughout pregnancy; and Control Group, consisting of women who would not carry out any physical exercise during pregnancy. The intervention consisted of the regular and moderate practice of water aerobics for 50 minutes three times a week in an indoor swimming pool with water at 28–30°C, initiated after enrollment and continued up to delivery [20]. The moderate intensity of exercises during the sessions was assured by monitoring patients' heart rate using a heart rate monitor [21] and kept around 70% of one's predicted maximun heart rate [4].

The following variables were evaluated in the two groups: age, parity, previous abortions and Cesarean sections, education level, gestational age at delivery, preterm birth rate, type of delivery, vitality of the newborn infant, weight of the newborn infant, low birthweight rate and birthweight adequate for gestational age. Other variables measured were: maternal weight gain, body mass index (BMI), percentage of non-fat and fat mass (using an adipometer – Lange^® ^to measure skinfold thickness). These variables were evaluated three times during pregnancy: 1) between 18 and 20 weeks; 2) between 22 and 26 weeks; and 3) between 32 and 36 weeks of pregnancy, at the same time when physical fitness was also evaluated [20]. In the water aerobics group, data were also collected on resting maternal heart rate, blood pressure and post-exercise condition.

The group sessions were carried out under the supervision of a trained instructor following a protocol [4]. Considering the low socio-economic status of the women participating in the water aerobics program, they received reimbursement of transportation costs between their home and the fitness center, and a kit containing the material required (swim-suit, bathing hat, robe, towel, bag and slippers).

At the time of delivery, women were admitted at the maternity hospital of the institution, under the care of the routine hospital staff, according to the regular schedules and procedures of the institution. A member of the research team was then informed and he/she attended labor and delivery to collect the data required by the study protocol. During delivery, additional data were collected, such as requesting analgesia during labor and delivery, and on the influence of immersion in water during water aerobics on the volume of amniotic fluid. These data were already published [20,22].

Women were discontinued if they abandoned the procedures required by the study protocol, including the water aerobics sessions and/or periodic evaluations. However, since the approach of intention-to-treat analysis was used, the information relating to discontinued subjects was included in the final analysis of results.

For the statistical analysis, distribution of control variables was initially evaluated to verify comparability of the two groups. A bivariate analysis was carried out in which qualitative variables were compared using the χ^2 ^test or Fisher's exact test and quantitative data was analyzed using Student's t-test. For the main results, risk ratios and their respective 95% confidence intervals (95% CI) were calculated. For the repeat variables over time (percentage of body fat and non-fat mass), MANOVA multiple analysis was used with Wilk's Lambda statistical test to evaluate the statistical significance of the differences according to time and group. Friedman's non-parametric multiple analyses were used to evaluate BMI. Significance was established at 5% and the software programs Epi Info, version 6.0, and SAS were used for analysis procedures.

## Results

The study started in March 2002 and terminated in November 2004. Of the 78 pregnant women initially eligible for participation, 7 were excluded (4 because they had great difficulty attending the water aerobics sessions regularly, 1 because of early fetal death diagnosed by ultrasonography, 1 because of fetal malformation revealed by ultrasonography, and 1 because of morbid obesity). Therefore, 71 pregnant women were randomized, 34 to the water aerobics group and 37 to the control group. By the time of the second evaluation, 9 women in the water aerobics group and 5 in the control group had discontinued. By the third evaluation, another 4 and 5 women had discontinued from the water aerobics and control groups, respectively. All discontinuations in both groups occurred because the women were unable to participate in the water aerobics program or attend the evaluation sessions, with the exception of one woman in the water aerobics group who was discontinued because of lost-to-follow-up. Data on her delivery and on the newborn infant are not available. The flowchart of study participants, as well as the demonstration that the two groups were similar with respect to the general characteristics of the women enrolled, are already included in the first article [20]. A total of 835 water aerobics sessions were recorded for these, corresponding to a mean of 24.6 sessions per woman.

**Figure 1:**
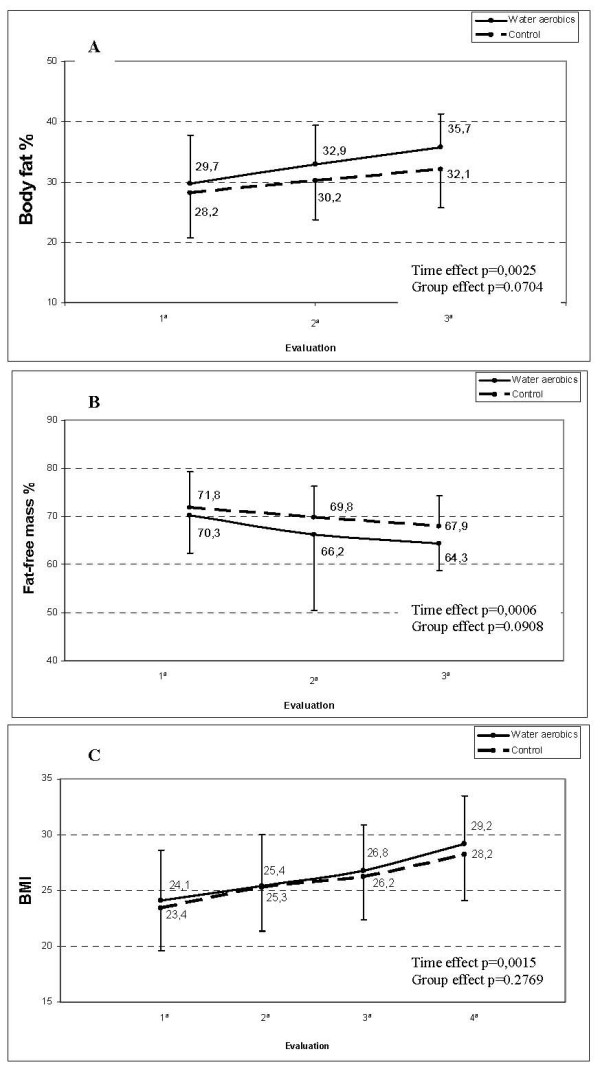
**Percentage of body fat (A), Percentage of fat-free mass (B) and Body Mass Index – BMI (C) during pregnancy according to group (water aerobics or control)**.

The weight gain of the women was similar in the two groups. BMI and the proportion of fat mass increased significantly during pregnancy, while the proportion of fat-free mass decreased significantly; however, there was no statistically significant difference between the two groups (Figure 1, Table 1). The groups were also similar with respect to gestational age at delivery, rate of preterm birth, low Apgar score at the first minute of life, weight at birth, low birthweight rate and birthweight adequate for gestational age. There were no cases of low Apgar score at 5 minutes. The occurrence of vaginal delivery was around 10% higher in the water aerobics group but this difference was not statistically significant (Table 2).

**Table 1:** Body composition during pregnancy according to group

**Body composition**	**Water aerobics**	**Control**	**p**
Weight (Kg) *			
Pre-gestational	63.8 ± 12.7	60.8 ± 10.2	0.27
At delivery	78.1 ± 14.7	75.9 ± 11.7	0.49
Mean difference	14.3 ± 2.1	15.1 ± 1.6	0.38
			
**Body Fat (%)^#^**			
1^st ^evaluation (18–20 weeks)	29.7 ± 8.0	28.2 ± 7.5	0.41
2^nd ^evaluation (22–26 weeks)	32.9 ± 6.5	30.1 ± 6.5	0.16
3^rd ^evaluation (32–36 weeks)	35.7 ± 5.6	32.1 ± 6.4	0.05
			
**Fat-free Mass (%)^@^**			
1^st ^evaluation (18–20 weeks)	70.3 ± 8.0	71.8 ± 7.5	0.42
2^nd ^evaluation (22–26 weeks)	66.2 ± 15.6	69.8 ± 6.5	0.28
3^rd ^evaluation (32–36 weeks)	64.3 ± 5.6	67.9 ± 6.4	0.05
			
**BMI^&^**			
Pre-gestational	24.1 ± 4.5	23.4 ± 3.8	0.48
1^st ^evaluation (18–20 weeks)	25.4 ± 4.6	25.3 ± 3.9	0.95
2^nd ^evaluation (22–26 weeks)	26.8 ± 4.1	26.2 ± 3.8	0.57
3^rd ^evaluation (32–36 weeks)	29.2 ± 4.2	28.2 ± 4.1	0.44

* Student's t-test

^# ^MANOVA (time effect p = 0.0025; group effect p = 0.0704)

^@ ^MANOVA (time effect p = 0.0006; group effect p = 0.0908)

^&^Friedman (time effect p = 0.0015; group effect p = 0.2769)

**Table 2:** Main maternal and perinatal outcomes according to group

**Characteristics**	**Water aerobics**	**Control**	**p**	**RR (95% CI)**
	n = 34	n = 37		
% vaginal delivery^#^	21/33	20/37	0.57	1.24 (0.73–2.09)
Preterm birth (% <37 weeks)^@^	2/33	3/37	0.56	0.84 (0.28–2.53)
Neonatal weight (X ± SD) in g*	3222.2 ± 562.7	3312.7 ± 656.1	0.54	-
Low birth weight (<2500 g)^@^	3/33	2/37	0.44	1.30 (0.61–2.79)
Adequacy of neonatal weight to Gestational Age^#^	29/33	29/37	0.46	1.50 (0.65–3.48)

* Student's t-test

^@ ^Exact of Fisher

^# ^χ^2^_Yates_

There were no statistically significant differences between the pre-exercise resting values and values of systolic and diastolic blood pressure and heart rate measured 3 minutes after the end of physical activity in the intervention group (Table 3).

**Table 3:** Evaluation of systolic blood pressure (SBP), diastolic blood pressure (DBP) and heart rate (HR) of pregnant women before and after water aerobics

**Parameters**	**Before water aerobics**	**After water aerobics**	p*
	n = 835	n = 835	
**SBP (mmHg)**	108.0 ± 14.8	108.9 ± 14.9	NS
**DBP (mmHg)**	68.1 ± 11.2	68.4 ± 10.5	NS
**HR (bpm)**	91.8 ± 10.6	92.2 ± 10.7	NS

* Student's t-test

## Discussion

Recent studies and recommendations indicate that the regular practice of moderate physical exercise may contribute towards a healthier pregnancy and that this practice, when adequately monitored, is not detrimental to the development of the pregnancy or the fetus [3,4,19,23].

The objective of the present study was to provide additional information on this subject, focussing on water aerobics as physical exercise during pregnancy. If on the one hand the present study failed to demonstrate any clear benefit to the mother practicing physical exercise in terms of a proportional reduction in fat mass, on the other hand, the safety of this practice is clearly demonstrated. There was no harm to the fetus/neonate in terms of vitality at birth, preterm birth, weight at birth, low birthweight rate or birthweight adequate for gestational age. Moreover, some indirect benefits may be considered, such as the greater occurrence of vaginal deliveries (around 10% greater, although this difference was not significant). Prevedel et al. [19] also found similar results with respect to maternal body composition, with a significant increase only in the rate of relative fat in the control group between the initial and final evaluations, and with respect to related preterm and inadequate birthweight for gestational age rates.

One of the main concerns with respect to the practice of aerobic exercise during pregnancy has been the possible greater occurrence of preterm associated with this practice. Indeed, the systematic review of data available on the subject reveals an estimated risk of preterm delivery attributed to aerobic exercise of 1.82 (95% CI 0.35 – 9.57) [14]. Combining all the data available with respect to the practice of water aerobics alone, including data from the present study, this relative risk may be estimated at 1.75 (95% CI 0.44 – 2.04), which strengthens the evidence on the safety of this practice during pregnancy.

The results of the present study may therefore contribute towards justifying the need to elaborate programs of moderate, water-based physical activity for sedentary, low risk pregnant women. This kind of physical exercise should improve the quality of life of the expectant mother, while providing her with an opportunity to participate actively in society, and may also reduce the occurrence of medical problems that result in lost working days.

The practice of physical exercise during pregnancy, mainly in water, seems to help reduce lower back pain in expectant mothers [10]. Women practicing water aerobics reported relief from lower back pain; however, no specific systematic evaluation of this complaint in the two groups of women in this study was carried out throughout the entire follow-up period, preventing us from drawing definitive conclusion.

The main limitation of this study is probably related to the difficulties the women had in regularly attending the scheduled water aerobics sessions. On the other hand, this may represent a more realistic picture of the possibilities involved in recommending a program such as this for sedentary women, even those with low-risk pregnancies, especially in under resourced settings. The women reported various kinds of difficulties and impediments such as household duties, commitments with children, husbands prohibiting their wives to attend classes, job-imposed limitations to schedule, embarrassment at having to wear a swimsuit or to use the fitness club pool together with the other women, lack of transportation, distance between the woman's home and the fitness club, and fear of water based on no previous experience. These difficulties were responsible for a relatively high rate of discontinuation in our study, leading to a situation where the sample size for the second and third evaluation of body composition was smaller than previously calculated, and could imply that the study is underpowered to draw firm conclusions. However, these are relatively new outcomes using a randomized controlled design and perhaps the current results could be combined with others already available [19] to draw a more powerful conclusion

If the majority of these impediments could be overcome by making it easier for women to exercise in a suitable pool at a convenient location near the maternity hospital or in community centers, by making sure that transportation is available, providing convenient exercise schedules and trained instructors to counsel women, With more women participating in these exercise programs, wider-ranging studies could perhaps then be carried out to clarify the outstanding issues with respect to the practice of physical exercise during pregnancy.

## Conclusion

Up to this time, no scientific data have been published showing any concrete detrimental effect to the mother or the fetus of moderate, water-based physical exercise, and the results of the present study again confirm previous data. In view of today's trend towards a more physically active society, the emphasis of these programs should be to assure the expectant mother that the state of pregnancy is not synonymous with confinement, and also that the practice of physical exercise, although gives no guarantee of a complication-free pregnancy, may result in a healthier pregnancy should a program of moderate physical activity be included as an integrated part of her prenatal care.

## Abbreviations

ACOG: American College of Obstetricians and Gynecologists; BMI: body mass index; CI: confidence interval; DBP: diastolic blood pressure; HR: heart rate; MANOVA: multivariate analysis of variance; RR: risk ratio; SBP: systolic blood pressure; UNICAMP: University of Campinas.

## Competing interests

The authors declare that they have no competing interests.

## Authors' contributions

SRC, JGC and RICP participated in all steps of the study, including research planning, data collection, analysis and writing the manuscript. EPB, ALB and CS participated in data collection and review of the manuscript. All authors gave suggestions, read the manuscript carefully, fully agreed on its content and approved its final version.
